# Cumulative effect of AOC1 gene variants on symptoms and pathological conditions in adult women with fibromyalgia: a pilot study

**DOI:** 10.3389/fgene.2023.1180777

**Published:** 2023-06-09

**Authors:** Gülşah Okutan, Teresa Perucho Alcalde, Eva Ruiz Casares, Bruno F. Penadés, Guerthy Melissa Sánchez Niño, Ana Terrén Lora, Sara López Oliva, Lorena Torrente Estríngana, Adriana Duelo, Ismael San Mauro Martín

**Affiliations:** ^1^ Research Centers in Nutrition and Health, CINUSA Group, Madrid, Spain; ^2^ VIVOLABS, Grupo Vivo, Madrid, Spain; ^3^ Faculty of Medicine, Permanent Training Center, Universidad Complutense de Madrid, Madrid, Spain; ^4^ Human Genetics and Molecular Diagnostics, Universidad San Pablo-CEU, Madrid, Spain; ^5^ International Institute of DAO Deficiency, Barcelona, Spain

**Keywords:** AOC1 gene, DAO gene, DAO deficiency, diamine oxidase, histamine intolerance, fibromyalgia, variant, single-nucleotide polymorphism

## Abstract

**Introduction:** The amine oxidase copper-containing 1 (AOC1) gene encodes for the diamine oxidase (DAO) enzyme. DAO is an enzyme that catabolizes some molecules, including histamine, and is the degradative enzyme in the polyamine catabolic pathway that is active in intestinal mucosal cells. Variants of AOC1 are associated with reduced DAO activity, resulting in accumulation of high levels of histamine and causing a wide range of neurological, gastrointestinal, and epidermal disorders, which are present in people with fibromyalgia. This study aimed to evaluate the impact of four AOC1 gene variants, namely, rs10156191, rs1049742, rs1049793, and rs2052129, on fibromyalgia symptoms measured by the Fibromyalgia Impact Questionnaire (FIQ), such as sleep disorders, atopic dermatitis, migraine, gastrointestinal (GI) disorders, allergies, and intolerances, in adult women with fibromyalgia.

**Methods:** The sample consisted of 100 unrelated women with fibromyalgia between 33 and 60 years of age (48.48 years ±7.35), whose were diagnosed by a rheumatologist based on symptoms such as pain, stiffness, and fatigue. Single-nucleotide polymorphisms (SNPs) of AOC1 were identified using oral mucosa samples collected following a standard hygiene protocol. DNA was extracted, and gene variants of interest were analyzed using multiplex single-nucleotide primer extension (SNPE). Clinical data were collected using the FIQ and a series of variables that quantified the intensity and frequency of the symptoms.

**Results:** The minor allele frequencies of rs10156191, rs1049742, rs1049793, and rs2052129 were 31.5, 10, 32.5, and 27%, respectively. Each variant was found to be in Hardy–Weinberg equilibrium, but partial linkage disequilibrium between AOC1 SNPs is suspected. The results show that fibromyalgia symptoms measured using the FIQ tend to increase with the number of risk alleles and that the intensity of dry skin and low stool consistency may be associated with an increase in the number of these alleles.

**Conclusion:** This study constitutes the first step in investigating associations between fibromyalgia symptoms and candidate variants of the AOC1 gene in DAO enzyme activity. Identification of reduced DAO activity may improve the quality of life and treatment of symptoms in fibromyalgia patients.

## 1 Introduction

Diamine oxidase (DAO) is an intracellular enzyme stored in the vesicular structures of the plasma membrane in various tissues, particularly in the intestinal mucosa. This enzyme catabolizes substrates such as diamines and histamine and acts as the degradative enzyme of the catabolic pathway of polyamines (Wolvekamp and de Bruin, 1994). Single-nucleotide polymorphisms (SNPs) in the DAO gene are associated with food allergies and inflammatory, gastrointestinal, and neoplastic diseases (Petersen et al., 2005). The DAO enzyme is encoded by amine oxidase copper-containing 1 (AOC1), which is expressed in intestinal and renal epithelial cells, where it is primarily stored in vesicles in the basolateral plasma membrane (Barbry et al., 1990; Schwelberger, 2004). The AOC1 gene is located on chromosome 7q36, and its most relevant nonsynonymous polymorphisms in Caucasian individuals include rs10156191 (p.Thr16Met), rs1049742 (p.Ser332Phe), and rs1049793 (p.His645Asp), which affect enzyme production of DAO (Ayuso et al., 2007), including rs2052129 (G4586T), which is found in the promoter region of the gene and produces lower transcriptional activity (Maintz et al., 2011). Noninvasive genetic analyses that identify SNPs associated with reduced DAO activity have been performed for years (Comas-Basté et al., 2020). In addition to these variants, recently, the SNP rs2052129, which possibly causes decreased transcriptional activity, has been shown to be associated with lower serum DAO activity (Maintz et al., 2006).

Numerous studies have been compiled reporting that a deficiency of DAO to catabolize histamine results in histamine intolerance (HIT) and that this can cause a wide range of neurological, gastrointestinal, or epidermal disorders (Comas-Basté et al., 2020). The ingestion of foods rich in histamine, alcohol, and histamine-releasing or DAO-blocking drugs can not only cause the characteristic symptoms of HIT but also genetic inhibition of the DAO enzyme that metabolizes histamine (Comas-Basté et al., 2020). This phenomenon has been underestimated, and its symptoms have been misinterpreted for a long time (Jarisch and Wantke, 1996; Smolinska et al., 2014; Kovacova-Hanuskova et al., 2015). In fact, it is held that approximately 1% of the population has HIT and that 80% of affected patients were middle-aged (Reese et al., 2017).

Histamine levels can also result in high levels in fibromyalgia patients, whose symptoms are closely related to the effects described and suggest that it may be a consequence of DAO deficiency. In general terms, fibromyalgia is a disease characterized by musculoskeletal pain accompanied by fatigue, stiffness, and a series of psychological disorders such as insomnia, headache, and anxiety (Dr Healthcare, 2022), which affects women more. Early diagnosis is essential for fast and personalized attention intervention; however, the detection of this disease has not been without complications due to lack of reliable markers for many years (Hackshaw, 2021). In this sense, the expression of DAO plays an important role in the diagnosis of HIT and, consequently, its concentration and activity can be used as a diagnostic method for fibromyalgia. In fact, DAO enzyme activity is considered a biomarker for diagnosing and applying new treatments for fibromyalgia (Dr Healthcare, 2022), and elevated histamine in the blood due to DAO deficiency in HIT patients can be used as a diagnostic tool but can be expensive and laborious (Zhao et al., 2022). Although there is still no specific biomarker to detect fibromyalgia, genetic testing may be a promising option (D’Agnelli et al., 2019). Following this detection tool, it has recently been observed that the prevalence of DAO deficiency in women with fibromyalgia was close to 75% based on the same SNPs that are presented here as a genetic test (Okutan et al., 2023). However, there is still no evidence on the combined effect of the variants on the characteristic symptoms of fibromyalgia.

Considering all the aforementioned points, this study aimed to evaluate the impact of four AOC1 gene variants, namely, rs10156191, rs1049742, rs1049793, and rs2052129, on fibromyalgia symptoms measured by the Fibromyalgia Impact Questionnaire (FIQ), such as sleep disorders, atopic dermatitis, migraine, gastrointestinal (GI) disorders, allergies, and intolerances, in adult women with fibromyalgia.

## 2 Material and methods

### 2.1 Sample selection

The study included 100 unrelated women with fibromyalgia aged 33–60 years, who were diagnosed by a rheumatologist based on symptoms such as pain, stiffness, and fatigue. The study excluded participants with multiple sclerosis, amyotrophic lateral sclerosis (ALS), Alzheimer’s disease, stroke, intellectual disability, tetraplegia, paraplegia, acquired immune deficiency syndrome (AIDS), cancer, and multiple chemical sensitivities, as these conditions may interfere with the results. Those already receiving DAO enzyme therapy or a low-histamine diet were also excluded. The participants were mostly Caucasian (95%); however, a small percentage of them were Latinas (4%) and Asian (1%). All participants were recruited by the Research Center in Nutrition and Health (CINUSA Group, Madrid, Spain) at the CINUSA Clinic and Ruber International Hospital (Paseo de la Habana 43, Madrid, Spain).

### 2.2 Genetic analysis

Four variants of the AOC1 gene, namely, rs10156191, rs1049742, rs1049793, and rs2052129, were genotyped. Oral mucosa sampling was performed by rubbing the inside of the cheek with a standard cotton swab and following a standard hygiene protocol. An automated isolation procedure on the QIAsymphony SP platform (QIAGEN) was used to isolate DNA from buccal mucosa samples. Genotyping of the samples was performed using multiplex single-nucleotide primer extension (SNPE), followed by capillary electrophoresis on an ABI 3500 Genetic Analyzer (Thermo Fisher Scientific) at VIVOLABS. The recommendations of the American College of Medical Genetics were followed to obtain the most accurate results (Richards et al., 2015).

### 2.3 Genetic DAO score

The genetic risk score was calculated as an estimate of the cumulative contribution of genetic factors to specific symptoms or conditions in patients with fibromyalgia. The coding of the genotypes of the four variants was estimated assuming that the alternative allele of each SNP modifies the risk of presenting or suffering greater intensity or frequency of symptoms. Thus, homozygotes for the reference allele were coded as 0, heterozygotes as 1, and homozygotes for the alternative allele as 2 for each variant, resulting in the sum of risk alleles. For tests involving alleles as categories, risk alleles were recoded as 3–4 and 5–6 in a category to increase the frequency of patients in each category.

### 2.4 Fibromyalgia Impact Questionnaire (FIQ)

The FIQ is a 10-item, self-administered questionnaire adapted to the Spanish language (Monterde et al., 2004). The first item (physical functioning) consists of 11 sub-items with four levels on a Likert scale. Item 2 (feeling well) indicates the number of days patients felt well in the last week. Item 3 (unable to go to work) and item 4 (work) indicate the number of days the patient was unable to work in the previous week and the difficulty of the work, respectively. The remaining six items are scored using visual analog scales, as in item 4, and assess pain, fatigue, morning fatigue, stiffness, anxiety, and depression. Scores from the items were adapted, recorded, and standardized on a 10-point scale. Thus, the highest scores represent a worse health status. The sum of the scores obtained by each participant constituted the FIQ score.

### 2.5 Clinical symptoms

A range of clinical symptoms related to sleep quality, atopic dermatitis (dry skin, urticaria, and eczema), migraine, and GI disorders (bloating, abdominal pain, burning, nausea, vomiting, and flatulence) were assessed (0–10), alongside the Bristol scale used to determine the consistency of stools (1-7). The frequency of GI disorders was evaluated using ordinal response variables (0 = never, 1 = a few times, 2 = sometimes, 3 = very often, and 4 = always), and scores were standardized at 10.

### 2.6 Statistical analysis

The relationship between FIQ Score and risk alleles was determined by multiple linear regression, adjusting for age and the relationship between the proposed symptoms. The activity of the AOC1 gene was estimated using logistic regression, calculating the odds ratio (OR) together with its 95% confidence intervals (CI) and adjusting for age using IBM SPSS Statistics 25.0 (IBM Corporation, Armonk, NY, United States). Hardy–Weinberg equilibrium (HWE) and linkage disequilibrium were evaluated using the *genetics* package (v1.3.8.1.3), and the Kruskal–Wallis test was used to compare the intensity and frequency of symptoms based on the risk alleles of the combined effect of the variants, correcting for type I error with the false discovery rate (FDR) in multiple comparisons, using the R statistical software in both cases (v4.1.2).

### 2.7 Ethical considerations

This study was approved by the Quirónsalud Catalunya (Barcelona, Spain) Pharmaceutical (CEIm) Hospital Group Research Ethics Committee (nº24/2021) and registered at ClinicalTrials.gov (identifier: NCT05389761).

This study was conducted using the latest versions of the Declaration of Helsinki (World Medical Association, 2013), GCP standards (ICH 2016 R^2^), and legal standards and regulations for biomedical research in humans (Law 14/2007 and RD 1090/2015).

Furthermore, the data were kept in strict compliance with the European Data Protection Regulation 2016/679 of the European Parliament and the Council of 27 April 2016, on the protection of data of natural persons (GDPR) and Law 3/2018 (LOPD).

All participants were invited to participate voluntarily, and only those who completed and signed the informed consent form were included in the study.

## 3 Results

### 3.1 Participants and genotype distribution

The sample consisted of 100 unrelated women between 33 and 60 years of age (48.48 years ±7.35) with fibromyalgia. The genotype distribution for each evaluated SNP was in Hardy–Weinberg equilibrium (*p* HWE >.05; Table 1), but linkage disequilibrium between SNPs was observed.

**TABLE 1:** Genotype distribution and minor allele frequencies of DAO SNPs.

SNP ID	Genotype distribution			*p* (HWE)	Minor allele	MAF
rs10156191	**CC**	**CT**	**TT**	.638	T	0.315
	0.46	0.45	0.09			
rs1049742	**CC**	**CT**	**TT**	.585	T	0.10
	0.80	0.20	—			
rs1049793	**CC**	**CG**	**GG**	.107	G	0.325
	0.49	0.37	0.14			
rs2052129	**GG**	**GT**	**TT**	.608	T	0.27
	0.52	0.42	0.06			

SNP, single nucleotide polymorphism; HWE, Hardy-Weinberg Equilibrium; MAF, minor allele frequency; A, adenine, C, cytosine; G, guanine and T, thymine.

### 3.2 Impact of fibromyalgia

**FIGURE 1:**
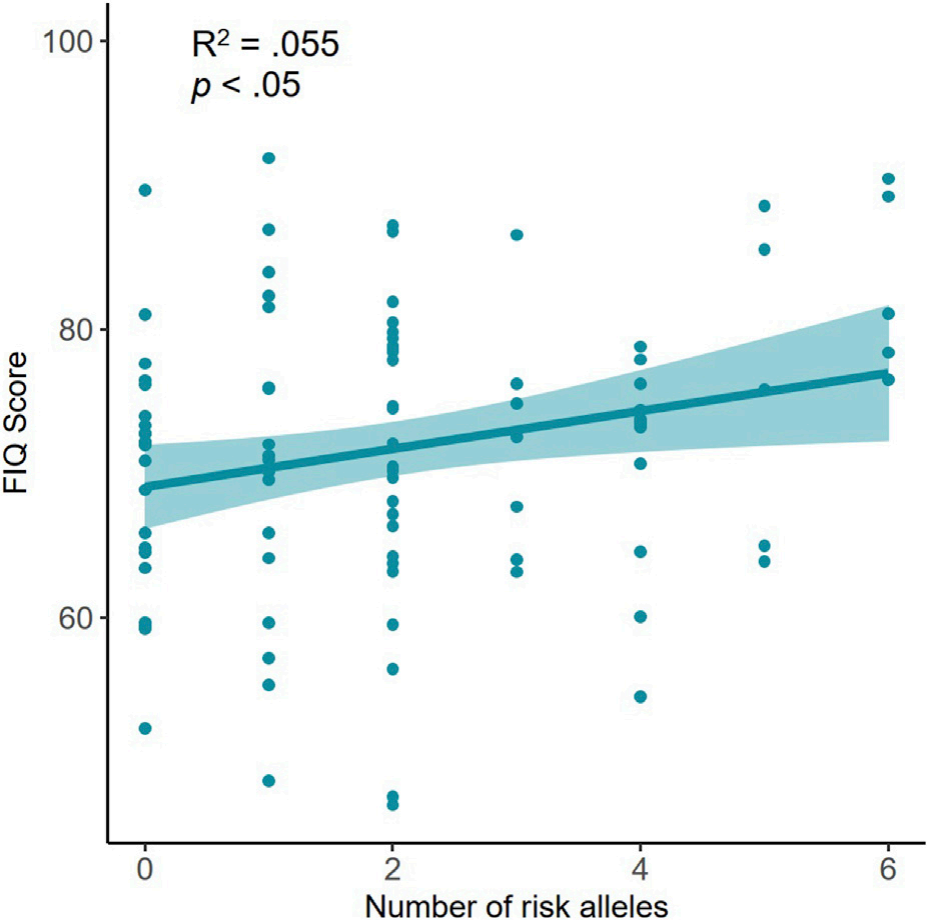
Cumulative effect of AOC1 gene variants rs10156191, rs1049742, rs1049793, and rs2052129 on FIQ Score. Contrast of the variable that includes the risk alleles adjusting for age.

The scores obtained by the participants in the items and the FIQ score are presented in Supplementary Table S1. The scores were compared as categories based on the number of risk alleles, and significant differences were only found in the item that assessed absence from work, although this value was not reliable, given the small number of patients who were working (Supplementary Table S2). Representative data were the FIQ score that was close to the significance level (*p* = .094), with the highest scores among patients with 5–6 risk alleles. This trend was tested by running a multiple regression analysis to predict the FIQ score as a function of the number of risk alleles, adjusting for the age of the patients, and a significant relationship was observed (*R* ^2^ = 0.055; *p* = 0.020; Figure 1).

### 3.3 Symptomatology

The presence, intensity, and frequency of the following symptoms were independently examined: sleep quality, atopic dermatitis, migraine, GI disorders, allergies, and intolerances. Most symptoms were widely displayed among women with fibromyalgia, including all who reported bloating and flatulence; however, a minority of participants reported allergies and/or intolerances (Table 2). Deficient DAO activity, which included participants with at least one risk allele, was not associated with any symptoms. Similarly, no association was observed when risk alleles were evaluated as numerical variables (Supplementary Table S3).

**TABLE 2:** Cumulative effect of the AOC1 gene variants rs10156191, rs1049742, rs1049793, and rs2052129 on the presence of insomnia, atopic dermatitis, migraines, GI disorders, allergies, and intolerances.

	Presence^a^ n	Normal DAO activity n (%)	Deficient DAO activity n (%)	OR (95% CI)	*p* ^b^
**Sleep quality**					
Insomnia	78	17 (73.9)	61 (79.2)	1.35 (0.44–4.13)	.594
**Atopic dermatitis**					
Dry skin	85	19 (82.6)	66 (85.7)	1.4 (0.38–5.13)	.609
Urticaria	79	15 (65.2)	64 (83.1)	2.5 (0.85–7.35)	.096
Eczema	50	9 (39.1)	41 (53.2)	1.8 (0.68–4.78)	.237
**Migraine**					
Headache	81	20 (87)	61 (79.2)	0.49 (0.13–1.95)	.314
**GI disorders**					
Bloating	100	23 (100)	77 (100)	—	—
Abdominal pain	96	23 (100)	73 (94.8)	—	.998
Burning	86	22 (95.7)	64 (83.1)	0.28 (0.03–2.36)	.244
Nausea	83	19 (82.6)	64 (83.1)	0.95 (0.27–3.37)	.934
Vomiting	51	12 (52.2)	39 (50.6)	1.05 (0.40–2.76)	.913
Flatulence	100	23 (100)	77 (100)	—	—
**Allergies**					
Drugs	17	2 (8.7)	15 (19.5)	2.17 (0.44–10.59)	.338
Gluten	20	5 (21.7)	15 (19.5)	0.69 (0.21–2.29)	.548
Food or pollen	27	7 (30.4)	20 (26)	0.70 (0.24–2.02)	.508
**Intolerances**					
Lactose	14	3 (13)	11 (14.3)	1.05 (0.26–4.32)	.941
FODMAP	5	1 (4.3)	4 (5.2)	1.71 (0.17–17.22)	.649

Note: Normal DAO activity included participants with zero-risk alleles for the four variants; deficient DAO activity included participants with 1–6 risk alleles. OR: odds ratio; CI: confidence interval.

^a^ The presence count for each symptom is equivalent to the percentage of participants.

^b^ Contrast of the variable that includes DAO activity adjusting for age.

**FIGURE 2:**
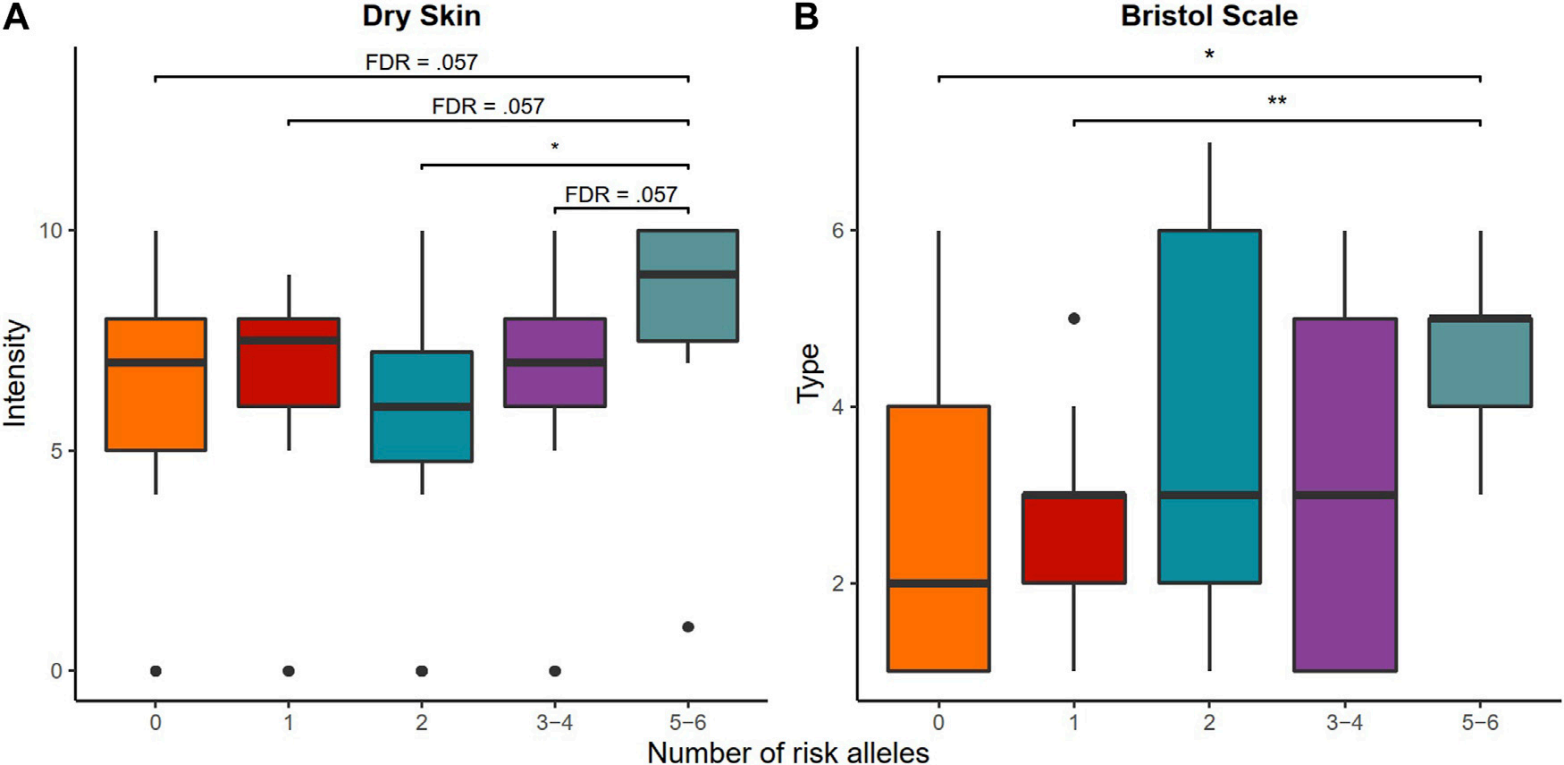
Cumulative effect of AOC1 gene variants rs10156191, rs1049742, rs1049793, and rs2052129 on **(A)** the intensity of dry skin and **(B)** Bristol scale. FDR: false discovery rate; significance value: FDR <0.05*; FDR <0.01**.

Dry skin and the Bristol scale scores showed differences between the risk alleles in terms of intensity (Table 3). In the first case, women with a higher number of risk alleles had a higher intensity of dry skin, typical of atopic dermatitis, than the group with only two risk alleles of the AOC1 gene (Figure 2A). Although the rest of the multiple comparisons with the groups formed by five and six risk alleles were not significant, they were borderline. In the case of the Bristol scale, the group of participants with the highest risk alleles presented less consistency in the stool compared to the participants with a single risk allele and to those with normal DAO activity who had a tendency to present with constipation (Figure 2B). There were no changes in the frequency of GI disorders according to the risk alleles (Supplementary Table S4).

**TABLE 3:** Cumulative effect of AOC1 gene variants rs10156191, rs1049742, rs1049793, and rs2052129 on intensity of symptoms related to quality of sleep, atopic dermatitis, migraines, and GI disorders.

Intensity	Genetic DAO score (number of risk alleles)					*p*
	0 (*n* = 23)	1 (*n* = 18)	2 (*n* = 29)	3–4 (*n* = 20)	5–6 (*n* = 10)	
Sleep quality						
Insomnia	4 (3-5)	4 (2-5)	3 (2-5)	3 (2-5)	3 (3-3)	0.575
Atopic dermatitis						
Dry skin	7 (5-8)	7.5 (6-8)	6 (4.5–7.5)	7 (6-8)	9 (7-10)	**< 0.05**
Urticaria	5 (0–7)	7 (5-7)	6 (5-7)	6 (4-7)	7.5 (6-9)	0.318
Eczema	0 (0–6)	0 (0–7)	4 (0–6)	5 (0–6)	2 (0–9)	0.600
Migraines						
Headache	7 (5.5–9)	9 (7-9)	8 (6-9)	6 (0–8.5)	7.5 (0–10)	0.228
GI disorders						
Bloating	8 (6-10)	8.5 (8-9)	8 (8-9)	9 (7-10)	8.5 (7-10)	0.657
Abdominal pain	7 (5-8)	7 (6-8)	7 (5-8)	7 (5–7.5)	8 (7-9)	0.425
Burning	7 (5-8)	5 (3-7)	5 (1-8)	6 (4.5–7)	7.5 (5-9)	0.134
Flatulence	8 (6-9)	8 (7-9)	8 (6-9)	8 (7-9)	9 (7-9)	0.871
Bristol scale (1-7)	2 (1-4)	3 (2-3)	3 (2-6)	3 (1-5)	5 (4-5)	**< 0.05**

Note: Medians and interquartile ranges are presented: Mdn (IQR).

Bold value indicates the significant values.

## 4 Discussion

This study addressed the combined effect of four AOC1 gene variants, rs10156191, rs1049742, rs1049793, and rs2052129, on fibromyalgia, measured using the FIQ, sleep quality, atopic dermatitis, migraine, GI disorders, allergies, and intolerances in adult women.

On a descriptive level, the minor allele frequencies of rs10156191, rs1049742, rs1049793, and rs2052129 were 31.5, 10, 32.5, and 27%, respectively. Although each variant is at Hardy–Weinberg equilibrium, partial linkage disequilibrium between AOC1 SNPs is suspected, as the frequencies of individuals carrying the association of DAO deficiency alleles are mostly higher than those expected, as calculated from frequencies of isolated genotypes. A conclusive analysis of linkage disequilibrium and the potential identification of haplotypes would require an increase in sample size.

The results suggest that the number of risk alleles tended to increase the impact of fibromyalgia, as measured by the FIQ. However, the deficit in DAO activity, which included at least one risk allele or evaluating the effect of the alleles as a numerical variable, was not associated with the presence of any symptoms, and only the intensity of dry skin and stool consistency could be explained by the highest number of risk alleles.

Despite the low number of symptoms associated with the four AOC1 gene variants, evidence suggests that reduced levels of the enzyme DAO, and thus high accumulation of extracellular histamine, are associated with symptoms of atopic dermatitis (Maintz et al., 2006; Maintz et al., 2011; Mušič et al., 2013; Schnedl et al., 2019), migraine (Maintz et al., 2006; Maintz et al., 2011; Schnedl et al., 2019), GI disorders (Raithel et al., 1999; Maintz et al., 2006; Maintz et al., 2011; Mušič et al., 2013; Schnedl et al., 2019), and food or drug allergies (Raithel et al., 1999; Maintz et al., 2006; Maintz et al., 2011; Schnedl et al., 2019), and that these symptoms may occur concurrently in many cases. It was demonstrated that administering 75 mg of pure liquid oral histamine under experimental conditions provoke immediate as well as delayed symptoms in 50% of healthy females without a history of food intolerance, including diarrhea, flatulence, and headache (Wöhrl et al., 2004). It was demonstrated that administration of pure liquid oral histamine under experimental conditions can cause immediate and delayed symptoms in healthy women with no history of food intolerance, such as diarrhea, flatulence, and headache (Wöhrl et al., 2004).

However, in the case of migraine, its association with low (Maintz et al., 2006; Maintz et al., 2011; Izquierdo-Casas et al., 2018; Schnedl et al., 2019) and high DAO activity has been reported (García-Martín et al., 2022). This is particularly important because previous studies that sampled people with migraine and analyzed the same variants assessed in this study observed that rs10156191 was associated with this disease only in women (García-Martín et al., 2015; Kaur et al., 2020) and the trend was considerably high, even though not statistically significant; however, it was not observed in men (García-Martín et al., 2022).

These studies highlight the role of the DAO enzyme in the body and the consequences of its deficiency and subsequent increase in extracellular histamine on human health. However, several diseases and symptoms have not been approached from a genetic perspective, much less by combining variants of the AOC1 gene associated with low DAO activity, to observe the predisposition to develop certain pathologies, such as fibromyalgia, when risk alleles accumulate.

### 4.1 Limitations

No association was observed between the presence of symptoms and genetic DAO deficiency, accounting for at least one risk allele, even as a quantitative variable. This may be due to the small sample size and its consequences on statistical power and other measures related to the intensity and frequency of the proposed symptoms. The fact that this is a cumulative variant model and the small number of participants with the maximum number of risk alleles increase the severity of the limitation. The study found no patients with the maximum number of risk alleles (seven). The homozygous recessive genotype rs1049742 is extremely rare in the population and was not observed in our sample. Furthermore, only a small number of patients had three, five, or six risk alleles, and it was decided to recode multiple alleles under the same category for intensity and frequency comparisons.

On the other hand, although it is true that no diagnostic tests were carried out to quantify serum/blood DAO or histamine activity and that this should not limit the aspirations of the study since genetic testing offers very promising results, the truth is that we do not know how it would have varied the results.

### 4.2 Conclusion

This study constitutes the first step in the search for AOC1 gene variants associated with fibromyalgia symptoms and low DAO enzyme activity. An increasing trend was observed between the effect of fibromyalgia and risk alleles and associations between dry skin severity and stool consistency when patients had a high number of risk alleles.

Addressing the cumulative effect of variants involved in the activity of the DAO enzyme through genetic testing and further studies with larger sample sizes and extended follow-up periods are required to confirm the findings of this study.

## Data Availability

The original contributions presented in the study are included in the article/Supplementary Material; further inquiries can be directed to the corresponding author.
