# Supplementary material for: Cumulative effect of AOC1 gene variants on symptoms and pathological conditions in adult women with fibromyalgia: a pilot study
Source: Front Genet. 2023 Jun 9;14:1180777. doi: 10.3389/fgene.2023.1180777 (PMC10288193; doi:10.3389/fgene.2023.1180777)
Supplement: Supplementary file 1 [file Table1.pdf]

Supplementary Material

Cumulative effect of AOC1 gene variants on symptoms and pathological conditions in adult women with fibromyalgia: a pilot study

Gülşah Okutan, Teresa Perucho Alcalde, Eva Ruiz Casares, Bruno F. Penadés, Guerthy Melissa Sánchez Niño, Ana Terrén Lora, Sara López Oliva, Lorena Torrente Estríngana, Adriana Duelo, Ismael San Mauro Martín*

*** Correspondence:** Ismael San Mauro Martín, info@grupocinusa.es

1 Supplementary Tables

Table S1. Scores of the Fibromyalgia Impact Questionnaire (FIQ)

FIQ	M ± SD	Mdn (IQR)
Physical function	5.89 ± 2.23	5.8 (4.07–7.41)
Feel good	7.76 ± 1.19	8.57 (7.14–8.57)
Absence from work	5.75 ± 3.44	6.00 (2.00–10.00)
Work	8.03 ± 1.59	8.00 (7.00–9.00)
Pain	7.62 ± 1.69	8.00 (7.00–9.00)
Fatigue	8.95 ± 1.38	9.00 (8.00–10.00)
Morning tiredness	8.96 ± 1.31	9.00 (8.00–10.00)
Rigidity	8.59 ± 1.12	9.00 (8.00–9.00)
Anxiety	7.48 ± 2.28	8.00 (6.00–9.00)
Depression	6.79 ± 2.54	7.00 (5.50–9.00)
FIQ score	71.79 ± 9.67	72.68 (64.73–78.18)

Note. Mean and standard deviation (M ± SD), median (Mdn), and interquartile range (IQR) are presented. The scores obtained were adapted, recoded, and standardised out of 10. The highest scores represent a worse state of health. The sum of the scores obtained for each participant allowed for the calculation of the FIQ Score.

Table S2. Combined effect of AOC1 gene variants rs10156191, rs1049742, rs1049793 and rs2052129 on the Fibromyalgia Impact Questionnaire (FIQ)

FIQ	Number of risk alleles					*p*
	**0 (n = 23)**	**1 (n = 18)**	**2 (n = 29)**	**3-4 (n = 20)**	**5-6 (n = 10)**	
Physical function	4.33 (2.08-5.86)	4.44 (2.86-6.67)	5 (2.73-5.93)	3.33 (1.67-4.77)	3.03 (1.67-5.15)	.257
Feel good	9 (7-9)	8.57 (7.14-8.57)	8.57 (7.14-8.57)	8.57 (7.14-8.57)	8.57 (7.14-8.57)	.655
Absence from work	2 (2-4)	8 (6-10)	4 (2-10)	4 (2-6)	10 (10-10)	**< .05**
Work	8 (7.5-9)	8 (7-8)	8 (7-9)	8 (7.5-9)	9 (8-10)	.270
Pain	8 (6-8.5)	8 (7-9)	8 (7-8)	8 (7-9)	8.5 (7-10)	.782
Fatigue	9 (8-10)	9.5 (8-10)	9 (8-10)	9 (9-10)	10 (9-10)	.580
Morning tiredness	9 (7-10)	9.5 (9-10)	9 (8-10)	9 (9-10)	9.5 (9-10)	.784
Rigidity	9 (8-9)	8.5 (8-10)	8 (8-9)	9 (8-9)	9.5 (8-10)	.634
Anxiety	7 (5.5-9)	8 (6-9)	8 (6-9)	7.5 (7-9)	10 (8-10)	.220
Depression	7 (5.5-9)	7 (5-7)	8 (5-9)	7 (5-8)	7.5 (6-10)	.611
FIQ score	69.42 (61.2-74.22)	69.29 (63.26-81.15)	72.07 (67.31-75.3)	69.75 (64.81-72.9)	77.15 (67.55-83.81)	.259

Note. Medians and interquartile ranges are presented: Mdn (IQR).

Table S3. Combined effect of AOC1 gene variants rs10156191, rs1049742, rs1049793 and rs2052129 on the presence of sleep quality, atopic dermatitis, migraines, GI disorders, allergies and intolerances

	Presence[a] n	OR (95% CI)	*p*[b]
**Sleep quality**			
Insomnia	78	1.06 (0.8-1.4)	.688
**Atopic dermatitis**			
Dry Skin	85	1.11 (0.79-1.57)	.536
Urticaria	79	1.12 (0.84-1.51)	.435
Eczema	50	1.18 (0.93-1.49)	.167
**Migraine**			
Headache	81	0.77 (0.58-1.02)	.069
**GI disorders**			
Bloating	100	-	-
Abdominal pain	96	0.62 (0.36-1.08)	.090
Burning	86	1.03 (0.72-1.46)	.882
Nausea	83	0.99 (0.73-1.33)	.932
Vomiting	51	0.83 (0.65-1.05)	.115
Flatulence	100	-	-
**Allergies**			
Drugs	17	0.87 (0.62-1.22)	.425
Gluten	20	1.07 (0.8-1.42)	.672
Food or pollen	27	0.84 (0.63-1.11)	.214
**Intolerances**			
Lactose	14	1.1 (0.8-1.51)	.572
FODMAP	5	1.3 (0.81-2.09)	.280

Note. OR: Odds Ratio; CI: Confidence Interval.

[a] The presence count for each symptom is equivalent to the percentage of participants.

[b] Contrast of the variable that includes the number of risk alleles adjusting for age.

Table S4. Combined effect of AOC1 gene variants rs10156191, rs1049742, rs1049793 and rs2052129 on the frequency of GI disorders

**Frequency**	**Number of risk alleles**					***p***
	**0 (n = 23)**	**1 (n = 18)**	**2 (n = 29)**	**3-4 (n = 20)**	**5-6 (n = 10)**	
**GI disorders**						
Bloating	7.5 (6.25-8.75)	7.5 (7.5-10)	10 (7.5-10)	7.5 (7.5-10)	10 (7.5-10)	.211
Abdominal pain	5 (5-7.5)	6.25 (5-7.5)	7.5 (5-7.5)	6.25 (5-7.5)	7.5 (5-7.5)	.664
Burning	5 (2.5-5)	2.5 (2.5-5)	5 (2.5-7.5)	5 (2.5-7.5)	6.25 (2.5-10)	.259
Nausea	5 (2.5-5)	3.75 (2.5-5)	5 (2.5-5)	3.75 (1.25-6.25)	5 (2.5-7.5)	.874
Vomiting	2.5 (0-2.5)	2.5 (0-2.5)	2.5 (0-2.5)	0 (0-2.5)	0 (0-2.5)	.882
Flatulence	7.5 (7.5-7.5)	8.75 (7.5-10)	7.5 (5-10)	7.5 (5-10)	10 (7.5-10)	.469

Note. Medians and interquartile ranges are presented: Mdn (IQR). The symptoms were evaluated with ordinal response variables (0=Never; 1=A few times; 2=Sometimes; 3=Very often; 4=Always), whose scores were standardized on 10.
